# OCT Monitoring of Low Energy Long Pulse 1064 nm Nd:YAG Laser Treatment of a Hereditary Hemorrhagic Telangiectasia–Associated Vascular Anomaly

**DOI:** 10.1155/crdm/8810881

**Published:** 2026-10-13

**Authors:** Feben Messele, Sara Araghi, Cameron Zachary, Natasha Mesinkovska, Kristen M. Kelly

**Affiliations:** ^1^ Department of Dermatology University of California Irvine, 118 Medical Surge Irvine 92697-2400 California USA berkeley.edu

**Keywords:** 1064 nm, hereditary hemorrhagic telangiectasia, HHT, Nd:YAG, OCT, optical coherence tomography

## Abstract

Hereditary hemorrhagic telangiectasia (HHT) is an autosomal‐dominant disorder characterized by vascular lesions with variable treatment responses. Optical coherence tomography (OCT) can provide noninvasive bedside visualization of cutaneous vasculature and monitoring of treatment response. A 79‐year‐old female with HHT underwent three treatments for a vascular lesion on her right fifth digit that had been resistant to pulsed dye laser, using a long pulse 1064 nm neodymium‐yttrium aluminum garnet laser. At lower fluences (50–55 J/cm^2^), clinical improvement was achieved without adverse effects. OCT imaging performed before and after treatment demonstrated an 89% decrease in vascular density and shallower vascular plexus depth posttreatment. OCT can be used to objectively measure changes in vascular characteristics in response to laser treatment.

## 1. Introduction

Hereditary hemorrhagic telangiectasia (HHT), characterized by vascular dysplasia commonly presenting on the fingers and lips, is an autosomal dominant disorder predominantly arising from loss of function mutations in the endothelial BMP9‐10/ENG/ALK1/SMAD4 signaling pathways [1]. HHT lesions can be difficult to treat and may require multiple sessions; adverse effects of treatment can include ulceration and scarring [2, 3]. We present a case where an HHT‐associated digital vascular lesion, resistant to pulsed dye laser (PDL), was treated with a long pulse (LP) 1064 nm neodymium‐yttrium aluminum garnet (Nd:YAG) laser. Treatment effects were documented with photographs and optical coherence tomography (OCT) imaging (Vivosight, Michaelson Diagnostics, Kent, England). This case uniquely demonstrates clinical and OCT monitored‐improvement after treatment of an HHT lesion using lower 1064 nm Nd:YAG fluences, selected to avoid ulceration.

## 2. Case Presentation

A 79‐year‐old female with HHT (clinical diagnosis) and a history of BRCA2‐associated breast and fallopian tube cancer presented for treatment of scattered vascular lesions on the perioral region and hands (Figure 1A,B). She previously received treatments with PDL with good response, except for a resistant lesion of the right fifth digit, which finally responded after three treatments with LP Nd:YAG (parameters were 5 mm, 20 ms pulse duration, 50–55 J/cm^2^, cryogen spray cooling setting of 10–15 ms spurt with a delay of 10–15 ms and postcooling of 10 ms). OCT images of the treatment area were taken before each treatment session and an untreated vascular lesion served as control; after three treatments, the right digit lesion improved, showing reduced erythema (Figure 2, Table 1). OCT imaging demonstrated an 89% decrease in vascular density and shallower vascular plexus depth posttreatment. The patient reported mild, transient edema without purpura, or ulceration posttreatments. Afterward, she received a fourth treatment and reported additional improvement.

**FIGURE 1:**
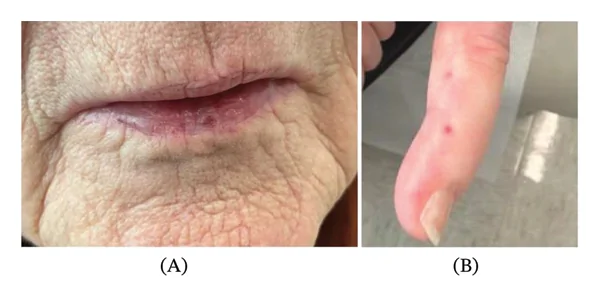
Telangiectasia on the mid vermillion lower lip (A) and the left fifth digit (B) demonstrating representative lesions seen in hereditary hemorrhagic telangiectasia.

**FIGURE 2:**
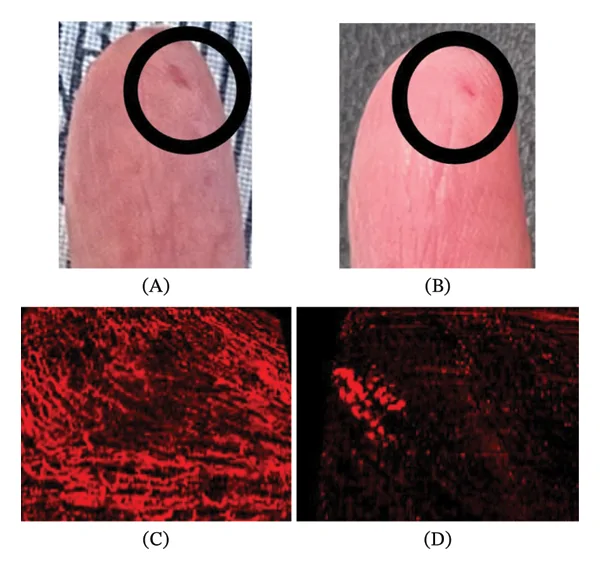
Resistant right 5th digit telangiectasia before (A, C) and after 3 Nd:YAG treatments (B, D), with optical coherence tomography images (C, D) demonstrating clinical improvement which correlated with reduced average vessel density and shallower superficial vascular plexus depth. (C, D: imaging area 6 × 6 mm).

**TABLE 1:** Superficial vascular plexus depth and mean vessel diameter and density pre‐ and post‐ three treatments.

	Before treatment	After 3 Nd: YAG treatments	Control
Plexus depth (um)	386	229	371
Density (%)	26	2.75	22
Diameter (um)	83	105	71

*Note:* Optical coherence tomography instrument determines plexus depth as the depth where vessel density reaches 50% of maximum vascular density. At this depth, vascular diameter (μm) and density (%) are calculated before treatment and after 3 Nd:YAG treatments. OCT imaging demonstrated an 89% decrease in vascular density and shallower vascular plexus depth posttreatment.

## 3. Discussion

OCT uses low‐coherence interferometry to provide high‐resolution cross‐sectional images of tissue, allowing visualization of skin microvasculature [4]. After laser therapy of this HHT‐associated vascular lesion, OCT demonstrated decreased vascular density and shallower plexus depth.

OCT has previously been used to assess HHT response to bevacizumab, showing a 36% reduction in vessel density [5]. Decreased density of telangiectasia on OCT has also been reported after 1064 nm Nd:YAG for non‐HHT‐associated telangiectasias [6].

Other studies have used Nd:YAG to treat HHT lesions without OCT monitoring. A comparison of dual wavelength sequential 595/1064 nm versus 1064 nm Nd:YAG alone in 26 HHT patients used Nd:YAG parameters of 3–7 mm, 15–20 ms pulse duration, and 90–225 J/cm^2^ with air cooling [3]. On a 5‐point‐scale, median improvement was 3 with Nd:YAG, with palpable scarring in one patient and transient edema in 12 patients [3]. Separately, 75%–95% clearance on digital lesions was reported after two treatments with 1064 nm Nd:YAG (parameters of 3 mm, 90 ms pulse duration, 200 J/cm^2^, and contact cooling) [7].

Our report used OCT to demonstrate an 89% reduction in vessel density after 1064 nm Nd:YAG laser therapy at lower fluences of 50–55 J/cm^2^ with clinical improvement and minimal to no adverse effects. Although laser parameters were not changed based on OCT input in this case, measurement of vascular diameter and depth could help guide parameter selection in future treatments. For example, OCT‐guided subpurpuric settings have been reported to successfully treat a resistant port wine capillary malformation [8].

Limitations of this report include single‐patient data, lack of genetic testing, and short follow‐up. This case demonstrates the potential of OCT to monitor changes in an HHT‐associated vascular lesion during treatment with LP Nd:YAG laser, achieving clinical and OCT‐measured improvement with minimal posttreatment side effects.

## Funding

Lutronic provided research grant and device.

## Consent

Patients gave consent for their photographs and medical information to be published in print and online and with the understanding that this information may be publicly available.

## Conflicts of Interest

The authors declare no conflicts of interest.

## Data Availability

The data that support the findings of this study are available on request from the corresponding author. The data are not publicly available due to privacy or ethical restrictions.
